# Glutamine Metabolism Is Essential for Stemness of Bone Marrow Mesenchymal Stem Cells and Bone Homeostasis

**DOI:** 10.1155/2019/8928934

**Published:** 2019-09-12

**Authors:** Tao Zhou, Yuqing Yang, Qianming Chen, Liang Xie

**Affiliations:** State Key Laboratory of Oral Diseases, National Clinical Research Center for Oral Diseases, Chinese Academy of Medical Sciences Research Unit of Oral Carcinogenesis and Management, West China Hospital of Stomatology, Sichuan University, 610041 Chengdu, Sichuan, China

## Abstract

Skeleton has emerged as an endocrine organ which is both capable of regulating energy metabolism and being a target for it. Glutamine is the most bountiful and flexible amino acid in the body which provides adenosine 5′-triphosphate (ATP) demands for cells. Emerging evidences support that glutamine which acts as the second metabolic regulator after glucose exerts crucial roles in bone homeostasis at cellular level, including the lineage allocation and proliferation of bone mesenchymal stem cells (BMSCs), the matrix mineralization of osteoblasts, and the biosynthesis in chondrocytes. The integrated mechanism consisting of WNT, mammalian target of rapamycin (mTOR), and reactive oxygen species (ROS) signaling pathway in a glutamine-dependent pattern is responsible to regulate the complex intrinsic biological process, despite more extensive molecules are deserved to be elucidated in glutamine metabolism further. Indeed, dysfunctional glutamine metabolism enhances the development of degenerative bone diseases, such as osteoporosis and osteoarthritis, and glutamine or glutamine progenitor supplementation can partially restore bone defects which may promote treatment of bone diseases, although the mechanisms are not quite clear. In this review, we will summarize and update the latest research findings and clinical trials on the crucial regulatory roles of glutamine metabolism in BMSCs and BMSC-derived bone cells, also followed with the osteoclasts which are important in bone resorption.

## 1. Introduction

Bone is a relatively dynamic organ which provides stiffness, shape, support, and locomotion for body structures [1]. It undergoes modeling and constant remodeling throughout life, exhibiting structure and shape changes. Bone modeling occurs from birth to adulthood and is responsible for gaining mass and changing the skeletal structure, as exemplified by the increases in bone length and diameter. Bone remodeling, tightly coupling bone resorption and formation, behaves the substitute for old tissues by new bones, thereby maintaining the mineral homeostasis and strength [2]. Osteoblasts for bone formation and osteoclasts for bone resorption are the main cells involved in bone remodeling; meanwhile, osteocytes derived from osteoprogenitors are also crucial in this biological process [3–6]. Recently, emerging evidences support that bone is an endocrine organ and manifests active metabolism, where cell bioenergetics plays an essential role in regulating intermediary metabolism [1, 7]. Collaboratively signaling networks contribute to an efficient transition in organisms between anabolic and catabolic states; thus, bone cells are capable to survive and grow in environments in which nutrient availability differs.

Virtually, biosynthesis requires amounts of exogenous fuel uptake, which can be converted to hydrolysis of adenosine 5′-triphosphate (ATP) inside the body to drive all cellular processes later [8]. The fuel sources containing glucose, free fatty acids, and the amino acids are excellent substrates for generating ATP in both cytoplasm and mitochondria through oxidative phosphorylation [9–11]. Their consumption and catabolism are adjusted automatically in order to match the distinctive energy demands in different stages covering proliferation, differentiation, and apoptosis, in which intracellular signaling molecules serve as checkpoints for fuel selection, storage, transport, and utilization [12]. In addition, extrinsic factors like glucocorticoids also change the fuel metabolism and biological behavior of bone cells in result [13].

Previous studies supported that glutamine metabolism as a regulatory node participated in many biological processes, including vessel formation, cancer progress, and immune regulation [14–16]. Recently, glutamine in bone homeostasis gained increasing concentration in mediating the proliferation, osteoblast, and adipocyte differentiation, immunological features of cell BMSCs [17]. Alternatively, the bioenergetics of osteoblasts, osteocytes, and even the adipocytes were also regulated directly or indirectly by glutamine metabolism, which were tightly related to the degenerative diseases such as osteoporosis. Mechanistically, it was elucidated that WNT/*β*-catenin signaling, mammalian target of rapamycin signaling (mTOR), hypoxia-inducible transcription factors (HIFs), and some other signaling pathways were involved in bone cell metabolic activities [18–20]. In this paper, we reviewed and updated the crucial regulatory roles of glutamine metabolism in BMSCs, BMSC-derived bone cells, and osteoclasts which expected to provide a novel therapeutic perspective for bone destructive disorders.

## 2. Glutamine Metabolism

Glutamine, a nonessential amino acid (NEAA) composed of carbon (41.09%), hydrogen (6.90%), oxygen (32.84%), and nitrogen (19.17%), is mainly synthesized by the enzyme glutamine synthetase (GS) using glutamate and ammonia (NH_3_) as a source. As the most bountiful and flexible amino acid in the body, it represents about 20% of the total free amino acids pool in the blood and 40% to 60% of the total amino acid pool in certain tissues [21–23]. Glutamine is hydrolyzed by glutaminase (GLS) to ammonium-ion (NH_4_) and glutamate, the latter is subsequently transformed to *α*-ketoglutarate (*α*-KG) occurring as a transamination or a deamination [24]. Then, *α*-KG enters the tricarboxylic acid cycle (TCA cycle) to generate ATP through the production of nicotinamide adenine dinucleotide (NADH) and flavin adenine dinucleotide (FADH_2_) [25]. Rather than being a substitute fuel source in TCA cycle, glutamine is also used as substrate for constructing proteins and nucleotides [26]. The nitrogen from glutamine through the practice of aminotransferases maintains the degrees of numerous amino acid pools in the cell, as exemplified by more than 50% of NEAAs originated from glutamine are used in protein synthesis in cancer cells *in vitro* [27]. It is indicated that, in cancer cells, mutations in TCA cycle enzymes, fumarate hydratase (FH) and succinate dehydrogenase (SDH), or complexes of the electron transfer chain (ETC), such as complex I and complex III, could promote glutamine utilization. In other words, TCA cycle, FH, SDH, and ETC are involved in its participation of nonessential amino acid production. For example, taken in by the cell through a transporter, glutamine is deaminated to glutamate by cytoplasmic GLS1, transferred by SLC25A11 into the mitochondrial matrix, and converted into *α*-KG. Then, *α*-KG follows TCA cycle steps until oxaloacetic acid, which is then converted into aspartate by aspartate transaminase (GOT2) and exported into the cytoplasm, which is critical to both purine and pyrimidine biosynthesis. After that, Asparate may be transformed into asparagine and arginine. In addition, glutamate in cytoplasm could be converted into arginine and proline [28–30]. Besides, glutamine also powers fatty acid synthesis through reductive carboxylation [31].

Glutamine metabolism was first put forward in 1935 by Hans Krebs, who reported that the brain cortex and retina of vertebrates and the kidney of rabbits and Guinea pigs could synthesize glutamate into glutamine and hydrolyze glutamine to ammonium glutamate [32]. Developing over time, much is known about the importance of glutamine metabolism in pathological conditions. Some tumor cells utilized glutamine to provide both nicotinamide adenine dinucleotide phosphate (NADPH) and carbon for lipid and glutathione biosynthesis as well as nitrogen for nucleotide biosynthesis, which was essential in controlling oxidative stress and supporting proliferation [33, 34]. Moreover, glutamine metabolism is also critical for liver-to-pancreas transdifferentiation, mature adipocyte inflammatory responses, and immunological cell functions [35–37]. And glutamine metabolism impacted epigenetic states as well as genome organization via *α*-KG, eventually altered cellular differentiation decisions [38]. More than 30 years ago, Biltz et al. firstly reported an active consumption and metabolism of glutamine in isolated calvaria and long bones [39]; subsequently, the role of glutamine in bone has drawn increasing attention.

## 3. Glutamine Metabolism in BMSCs

BMSCs, known as nonhemopoietic multipotent mesenchymal cells, are traditionally capable to differentiate into osteoblasts, adipocytes, and chondrocytes, thereby regulating bone homeostasis [40–42]. Recently, the energy metabolisms including glucose metabolism, glutamine metabolism, and fatty acids in MSCs in various contexts are reported constantly [43–45]. Glucose is a major energy and carbon source for mammalian cells and has been known as a major nutrient for osteoblasts since the early 1960s [46]. Instead of energy supplement, aerobic glycolysis in osteoblasts may be linked with the citrate secretion, which plays a critical role in the formation of apatite nanocrystals in bone [47, 48]. Therapeutic strategies that target glucose metabolism tend to apply to patients diagnosed with systemic diseases such as type 2 diabetes mellitus and chronic kidney disease [49, 50]. Moreover, Thrailkill et al. suggested that treatment with insulin alone only partially corrected both hyperglycemia and diabetic bone phenotype in twelve-week-old diabetic mice, which means the therapy targets in other metabolism are required [51]. Fatty acids, generated from stored triacylglycerides or fat depots and released into the circulation, are degraded in the mitochondria for the generation of ATP in bone cells, while the amount that is utilized for ATP production is currently unknown [52]. Similar to fatty acids, the extent that amino acids contributes to oxidative phosphorylation remains unclear at present; however, there are increasing numbers of researches on glutamine. Glutamine as the second critical regulator after glucose exerts an essential modulation in BMSC proliferation, lineage allocation, osteoblast specification, and even the immunomodulatory properties.

### 3.1. Glutamine Metabolism in BMSC Proliferation

With regard to the proliferation of cells, Eagle et al. initially described the importance of glutamine in cell proliferation *in vitro* and meticulously essential for MSC proliferation [53]. In synchronized HeLa cells, glutamine, as well as glucose, is required for progression through the restriction point in mid-to-late G1. And glutamine is the only essential substrate for the progression through S phase into cell division, which was also indicated by combining pulse-chase LC-MS-based isotope tracing with computational deconvolution and metabolic flux modeling in synchronized cell populations [54, 55]. Mechanistically, glutamine has been reported to progress through the restriction point in mid-to-late G1 as well as exit S phase that was efficient for cell division beginning [54]. It is indicated that glutamine could enhance the expression of cyclin D1 and D3 and regulate cyclin-dependent kinase (CDKs) that were able to promote the passage into S phase and downregulate p21 expression, a key regulator for the cycle checkpoint of G1/S [56]. And this phenomenon may be associated with GLS, since glutamine increased the activity of GLS and glutamate dehydrogenase (GDH) through the mTOR/S6 and MAPK pathways in a dose-dependent manner, which finally promoted the cell proliferation [57]. However, the concrete mechanism remains unclear currently. In addition, it is commonly accepted that glutamine provided precursors for downstream synthetic steps, such as the DNA replication in S phase and lipid synthesis in G2 phase. And the majority of TCA carbons and nitrogen of some NEAAs derived from degraded glutamine in endothelial cells [15, 58]. Glucose is a major energy and carbon source for mammalian cells and has been known as a major nutrient for osteoblasts since the early 1960s. What is more, glutamine also provides a small amount of energy, since the glutamine-consuming enzymes are found largely in mitochondria and far from the primary need for ATP. Additionally, Karner's group found that BMSC proliferation and colony expansion were largely correlated with amino acid transaminase-dependent *α*-KG production, which could partially explain the negative impact of reduced GLS activity on BMSC proliferation [17]. However, the contribution of other amino acid biosynthesis derived from glutamine metabolism to BMSC proliferation has not been clear yet [33, 59]. For tumor cells in other tissues, glutamine satisfies biosynthetic and bioenergetic demands of these cells via anaplerotic entry to the TCA cycle and reductive carboxylation, thus regulating cell survival and proliferation [60, 61]. In contrast, the proliferation rates of skeletal progenitor cells seemed less important connection with glutamine-dependent reductive carboxylation or TCA cycle anaplerosis, which suggested distinctive roles of glutamine metabolism in different types of cells [19].

### 3.2. Glutamine Metabolism in BMSC Differentiation

**Figure 1:**
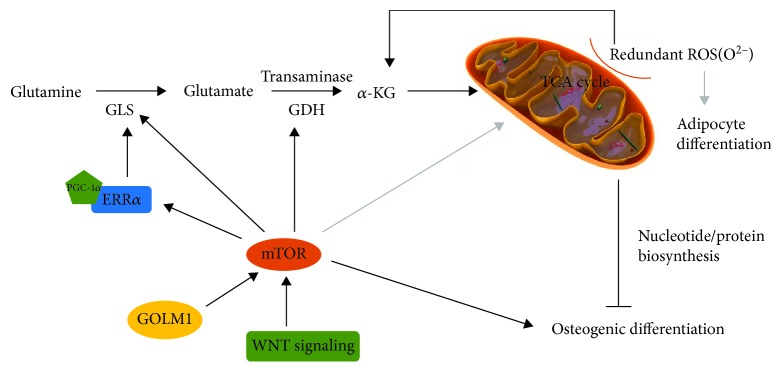
Glutamine-dependent regulation of BMSC osteogenic and adipocyte differentiation. The black arrows represent the signaling pathway in osteogenic differentiation regulated by glutamine; meanwhile, the gray arrows represent the signaling pathway in adipogenic differentiation.

Osteogenic and adipocyte differentiations are the pivotal linage commitments of BMSCs in skeletal development. BMSCs consume and metabolize a significant amount of glutamine as they undergo differentiation into the osteoblast but not the adipocyte lineage. As BMSCs differentiated toward osteoblasts, glutamine metabolism provided ATP through the TCA cycle with a declined contribution to citrate [17]. Furthermore, an integrated mechanism in a glutamine-dependent pattern was involved to meet energetic and synthetic demands during BMSC differentiation (see Figure 1).

#### 3.2.1. GLS

Mitochondria is a pivotal place covering many complex metabolic reactions [62]. GLS catabolizes glutamine into glutamate, then *α*-KG, which replenishes anaplerosis of TCA intermediates to maintain mitochondrial activity and supply metabolic intermediates for active biosynthesis in osteogenesis [63]. Experimental evidence suggested GLS as the targeted enzyme of glutamine metabolism which influenced the differentiation of BMSCs. Genetically inhibiting glutamine metabolism via deletion of *Gls* in BMSCs resulted in reduction of overall osteoblast numbers and capability of bone information, consequently causing decreased bone mass relative to wild-type littermates [17]. Alternatively, miRNA as an important regulator was able to establish a complex circuit in bone homeostasis by interacting with different genes [64]. Recent evidences reported that miRNA-206 participated BMSC bioenergy by directly bounding to the 3′-untranslated region (3′-UTR) of GLS mRNA, which resulted in the suppression of GLS expression and glutamine metabolism and eventually inhibited the osteogenic differentiation of BMSCs [65].

#### 3.2.2. mTOR

mTOR is a sensor of growth factors whose activation increases bone width and mass as a result of hyperproliferated MSCs but declines bone length and mineral contents due to defective MSC differentiation [66]. Mechanistically, mTOR is a central target of intrinsic control in bone cells which integrates various molecules associated with glutamine metabolism in BMSC differentiation. Previous studies indicated WNT signaling influenced osteoblasts biological behaviors by enhancing both cell numbers and protein synthesis activity [67]. Importantly, to meet the increased energetic and synthetic need, the anabolic mechanism directly responded to WNT signaling to impact osteoblast differentiation of BMSCs. WNT signaling targeted the mammalian target of rapamycin complex1 (mTORC1) to stimulate glutamine entry to the TCA cycle, subsequently it lowered intracellular glutamine levels. Then, the general control nonderepressible 2-mediated (GCN2-mediated) with integrated stress response (ISR) pathway was triggered due to the WNT-induced reduction of glutamine, which stimulated the expression of genes that responsible for amino acid transport, tRNA aminoacylation, and protein folding [68]. Previous studies also suggested that mTORC1 activation stimulated glutamate to *α*-KG conversion by activating GDH, thus promoting cancer cell proliferation [69]. The activation of mTOR signaling pathway stimulated by Golgi membrane protein 1 (GOLM1) overexpression in BMSCs was in sympathy with that in cancer cells, behaving inhibited osteogenic differentiation of BMSCs due to increased GDH activity and glutamine to *α*-KG conversion. [20].

#### 3.2.3. ERR*α*

Estrogen-related receptor *α* (ERR*α*), an orphan nuclear hormone receptor, is capable of regulating the transcription of related genes. Previous studies reported that ERR*α* positively regulated adipocytic and chondrocytic differentiation of MSCs while behaved a dual effect on osteoblast differentiation in Runx2- and/or WNT-target manner [70]. Recently, the age-related restriction of BMSCs has been reported as an essential factor in bone degenerative progress because of declined osteogenic capacity and unbalanced lineage allocation [71, 72]. And the dynamic expression patterns of ERR*α* with ages were tightly associated with BMSC osteoblast differentiation. A study displayed that ERR*α* expression was obviously reduced in elder rats, which was consistent with the deteriorated osteogenic capacity with ages [73]. Besides, ERR*α* was dysregulated in age-prevalent diseases like osteoarthritis and rheumatoid arthritis [74, 75]. As for cell level, ERR*α* reached peak protein expression levels at early phase of osteoblast differentiation and declined at mineralization stage while mRNA levels remained stable. It indeed supported the view that ERR*α* was inactivated after the onset of osteoblast maturation and it regulated osteoblast differentiation in a time-specific manner [76]. However, precise molecular mechanism remains unclear. Dysregulation of mitochondrial function is a common feature of aging, and coactivation of ERR*α* with proliferator-activated receptor gamma coactivator 1-*α* (PGC-1*α*) regulated mitochondrial biogenesis through fatty acid oxidation and energy expenditure related to ROS [77, 78]. Through binding to its promoter, ERR*α* directly regulated GLS expression, leading mitochondrial Gln-dependent anaplerosis critical to TCA cycle and biosynthesis of nucleotides and proteins. Aging negatively impacted on this ERR*α*/GLS signaling pathway, and repaired ERR*α* and GLS expression could partially restore osteogenic capacity of MSCs to resist bone loss [73]. In addition, the synthesis master regulator mTOR modulated ERR*α*/GLS signaling via affecting ERR*α* transcriptional activity, which may be a targeted therapy for aging-related bone loss [73].

#### 3.2.4. ROS

Reactive oxygen species (ROS) originate from the oxidation of metabolic intermediates of ETC and are usually produced in the form of superoxide in the mitochondria [79]. The complexes of the respiratory chain in mitochondria are the main ROS production sites, especially complexes I and III. Besides, many other proteins such as pyruvate dehydrogenase (PDH) and electron transfer flavoprotein (ETF) are also ROS producers [80]. ROS are not only a consequence of differentiation but also are critical components of pathways regulating stem cell differentiation [81]. They are precisely regulated to prevent oxidative damage of cells in normal circumstances; elevated ROS in BMSCs with ages were reported to destruct the lineage allocation, displaying promoted adipogenesis and blocked osteogenesis [82, 83]. A potential mechanism may be the toxic accumulation of *α*-KG under excessive oxidative metabolism. Increased activity of PDH and loss of mitochondrial membrane potential (MMP) with a transformation to TCA cycle most likely enhanced pyruvate entry into mitochondria, thus accumulating toxic metabolites [84]. Then, it resulted in nucleocytoplasmic vacuolation and chromatin condensation which obviously prevented osteogenic and adipocyte differentiation. Simultaneously, the accompanying DNA damage, inhibition of histone H3 (Lys27) of acetylation, and increased HIF-1*α* degradation contributed to the death of BMSCs [84]. Moreover, another study reported that increased glutathione content from glutamine was important to offset the detrimental effect of ROS to the osteoblast fate [17]. Alternatively, compared with the positive role of ROS, glutamine was less chief in adipocyte differentiation of BMSCs. The mitochondrial-generated ROS enhanced adipocyte differentiation in a mTORC1-dependent pattern, which could explain the phenomenon that neither glutamine consumption nor GLS activity altered during adipocyte differentiation relative to undifferentiated BMSCs [85, 86].

### 3.3. Glutamine Metabolism of BMSCs in Osteoimmunology

Apart from the self-renewal and multilineage differentiation features, MSCs are known to exert an immunosuppressed modulation by expressing adhesion molecules and secreting effectual factors like cytokines, chemokines, and growth factors [87–89]. Researches about glutamine metabolism in immune system in recent decades also prompted the recognition of its regulatory role in adaptive immunity and innate immunity, covering lymphocytes, neutrophils, and macrophages as well as a series of cytokines [90–92]. In BMSCs, the concentration of glutamine was relevant to their immunology properties. High dose of glutamine displayed an enhancement for immunosuppressive properties of BMSCs via affecting inflammatory cytokines, displaying decreased levels of proinflammatory cytokines like interleukin-1*β* (IL-1*β*) and IL-6 and increased levels of anti-inflammatory cytokines IL-10, and transforming growth factor-*β* (TGF-*β*) [93]. Mechanistically, varied production of proinflammatory cytokines may relate to the reduced expression of phosphorylated nuclear factor kappa-B (NF-*κ*B) and high level of signal transducer and activator (STAT-3) in BMSCs as they control cytokine production [94]. Additionally, IL-10 was critical in immune responses in glutamine concentration as they inhibited activation of NF-*κ*B, thus modulating the cytokine production. Meanwhile, both IL-10 and STAT-3 increased in BMSCs with glutamine, which could be explained that the anti-inflammatory effects of IL-10 were mediated by STAT-3, and in turn, IL-10 was also reported to promote STAT-3 to reduce amounts of proinflammatory cytokines [95, 96]. Additionally, the proliferation of lymphocytes and macrophages was inhibited when cocultured with BMSCs in glutamine medium, both followed with an increased production of IL-10 [97]. The increased IL-10 may be attributed to the transformation of macrophages to an anti-inflammatory M2 phenotype with the induction of MSCs [98], whereas the precise mechanism of immunomodulation in BMSC-mediated glutamine is unclear.

## 4. Glutamine Metabolism in Osteoblasts

Characterized as the chief bone-making cells, osteoblasts take charge of producing large amounts of both collagen I-rich bone matrix and ectoenzymes controlling matrix mineralization. They follow timely programmed steps and express specific genes under the control of proosteogenic pathways. WNT signaling pathway is pivotal to promote the commitment towards an osteo/chondroprogenitor of BMSCs, especially in the early steps in osteoblast differentiation [99]. It is suggested that WNT signaling directly reprograms cellular metabolism in osteoblast lineage cells by stimulating aerobic glycolysis, glutamine catabolism, and fatty acid oxidation [67]. Additionally, glutamine catabolism has been identified as a crucial regulatory step in satisfying both energetic and synthetic requirements which is connected with WNT-induced bone anabolism in immature osteoblasts.

Karner et al. reported that glutamine was both an energy source and a protein-translation rheostat which was responsive to osteoblast differentiation [68], and impaired osteoblast differentiation with ages in BMSCs may be linked with declined glutamine consumption [73]. Meanwhile, Brown et al. supported that glutamine significantly improved osteoblast viability and enhanced the utilization of glucose in both human osteoblast-like cell lines and mouse calvarial osteoblasts, and higher levels of osteocalcin expression were beneficial for matrix mineralization [100]. Furthermore, considering that glutamine directly stimulated collagen type1a1 transcription in fibroblasts, the practice of glutamine on mineralization in osteoblast cultures might be owing to an influence on collagen expression [101]. However, it remains unknown whether glutamine anaplerosis is required for physiological osteoblasts activity in bone formation due to the lack of systematic analyses in osteoblasts with *Gls* depletion [102].

## 5. Glutamine Metabolism in Chondrocytes

The commitment of BMSCs to the chondrogenic lineage is a significant event to initiate the endochondral ossification that BMSCs firstly give rise to immature chondrocytes and cartilage primordia. Integrated signaling among growth factors and components of the extracellular matrix containing collagens, proteoglycans, glycosaminoglycans (GAGs), and proteases regulate chondrocytes collaboratively to facilitate progressive changes in endochondral ossification and bone formation [103]. Glutamine was initially shown to sustain glycosaminoglycan and protein synthesis as a carbon and nitrogen provider in extracellular matrix metabolism in chondrocytes [104]. In view of the special avascular environment of cartilage, it was widely assumed that cells within cartilage were hypoxic and hypoxia regulated the energetic state of maturing cells [105]. However, an excessive hypoxic environment was harmful for chondrocytes, and it was usually followed with a reduced utilization of glutamine and declined content of glutathione, which was possibly attributed to the downregulated mitochondria1 function and inhibited oxidative deamination [105]. HIF-1*α* is a protein expressing in hypoxic microenvironment, and higher expression of HIF-1*α* under hypoxic condition is of great necessity for chondrocytes survival in an intrinsic mechanism [106, 107]. As HIF-1*α* mediated an upregulated expression of GLS1, the flux of glutamine to *α*-KG was enhanced to favor *α*-KG-dependent proline and lysine hydroxylation of collagen, and it was beneficial to increase bone mass by endowing the resistance of the cartilaginous matrix to protease-mediated degradation [108]. In some pathological situations, glutamine also exhibited a protective effect on chondrocytes. For instance, glutamine upregulated glutathione concentration in chondrocytes to protect cells from injury in surgery or infectious conditions [109, 110]. In stress conditions, glutamine exerted chondroprotective effect by enhancing the expression of heat shock protein 70 (HSP70), which reduced chondrocytes apoptosis to prevent the progress of cartilage degeneration [111]. Importantly, two energy-dependent anabolic processes collaboratively regulated the biological behavior in chondrocytes. The imbalance of glucose-mediated reduced collagen synthesis and glutamine-mediated increased bone mass in chondrocytes will lead to the skeletal dysplasia [108].

## 6. Glutamine Metabolism in Osteoclasts

To maintain skeletal architecture and strength, a homeostatic balance between new bone formation and old or damaged bone resorption is required. Osteoclasts derived from the hematopoietic lineage mainly degrade bone matrix and liberate the calcium and phosphate, eventually exhibiting regulation on bone mass as well as quality [112]. It was suggested that L-glutamine had a significant impact on early phase of osteoclast differentiation and maturation stage [113]. Following the uptake through SLC1A5, a Na^+^-dependent transporter of L-glutamine [114–116], osteoclasts converted glutamine to glutamate and then to *α*-KG, which was important as an anaplerotic substrate in osteoclast differentiation [117]. Additionally, glutamine was an essential fuel for the acquisition of bone-resorbing activity in mature, multinucleated osteoclasts [113]. Morten et al. reported that hypoxia stimulated glutamine consumption in osteoclasts, which was similar to SK–N–SH neuroblastoma and A549 lung adenocarcinoma cells [118, 119]. The increased glutamine uptake may mainly contribute to biosynthesis as glutamine withdrawal had no effect on either ATP production [61].

## 7. Therapeutic Potential of Glutamine in Bone Disorder Treatment

Energetic metabolism has gained improving attentions in the past decades for the regulation in the delivery and utilization of nutrients throughout the body, and the metabolic inflexibility is associated with various pathological process [120]. In updated clinical trials, amounts of researches have been arisen to elucidate the influence of glutamine in the improvement of adverse reactions induced by treatments and the potential applications in diagnosis (see Table 1). Additionally, glutamine is pivotal for both energy production and redox homeostasis in bone homeostasis, which can be a potential strategy in bone diseases such as osteoporosis and osteoarthritis.

### 7.1. Osteoporosis

Osteoporosis, mainly occurring in postmenopausal women and elder group, is characterized by low bone mass and deterioration of the bone microarchitecture which eventually behaves increased fracture susceptibility [151]. Previous researches reported aging-related changes of glutamine metabolism in osteoporosis could break the balance between osteogenic and adipocyte differentiation of BMSCs through key enzyme destruction in glutamine metabolism or mitochondria metabolic deterioration [73, 84]. Early anabolic therapies associated with glutamine may be a good way to treat osteoporosis from the perspective of etiology. Glutamine supplement (L-glutamine/L-alanyl solution (2.0 ml/kg) through the tail veins in the first 7 d was noted to obtain quicker and more regular primary callus and cartilaginous callus through attainments of positive nitrogen balance in standardized albino rats, which was instrumental in the healing of fractured osteoporosis patients [152]. However, the effect was tiny on enhancing the quality of fracture healing under conditions of stress, only exhibiting some influence on the speed of healing [152]. Virtually, glutamine precursor has been explored to apply in the treatment of osteoporosis in animal model. 2-Oxoglutarate (2-Ox), a precursor of glutamine, has been identified to promote the thickness of cancellous bone, growth plate, and articular cartilage in fundectomy-induced osteopenic bone [153]. It was also applied in osteoporosis induced by glucocorticoid treatment in premature infants with inflammatory and autoimmune disorders, which improved levels of growth hormone and osteocalcin concentration and preserved microarchitecture of trabecular bone [154].

### 7.2. Osteoarthritis

Osteoarthritis, characterized by degeneration of the articular cartilage and subchondral bone pathologically, is often diagnosed by the symptoms of pain, joint stiffness, and disability [155]. In osteoarthritis patients, inflammatory cytokines and ROS are induced by nonphysiological mechanical loading and heat stress facilitated by deviant joint movements, eventually contribute to the pathological progression. The treatment of chondrocytes with glutamine protected cells from heat stress and NO-induced apoptosis, thereby preventing osteoarthritis [111]. Fujita et al. indicated that heat stimulation and glutamine could stimulate the expression of HSP70 in rat articular cartilage *in vivo*, which may be involved in the suppression of osteoarthritis progression [156]. As stem cell-based therapy is a potential approach for osteoarthritis, researches about cellular metabolism in stem cells contribute to the application of cell-based treatment in general. Stegen et al. suggested that HIF-1*α*-mediated conversion of glutamine to glutathione synthesis was beneficial to maintain redox homeostasis under oxidative or nutrient stress, consequently exerting beneficial impact on cell survival [19]. The transplantation of adipose-derived mesenchymal stem cells (Ad-MSCs) in 1 ml of Dulbecco's modified Eagle's medium (DMEM) was injected into articular defect area of the osteoarthritis rabbits, and the overall healing score of experimental knees was superior when compared to the control group just received 1 ml of DMEM, in which 2 mM L-glutamine was included [157]. In addition, when it comes to osteoarthritis patients who received TKA, supplementation with a combination of *β*-hydroxy-*β*-methyl butyrate, L-arginine, and L-glutamine (HMB/Arg/Gln) during the postoperative recovery could suppress the loss of muscle strength [150].

## 8. Conclusion

Recent evidences indicated that glutamine is a critical regulator in bone homeostasis via supporting energy as a substitute carbon source through TCA cycle and providing precursors for protein and nucleic acid synthesis. At cellular level, glutamine metabolism mediate the bioenergy of bone cells including BMSCs, osteoblasts, chondrocytes, and osteoclasts, thus influencing their capabilities of the proliferation, differentiation, and mineralization. Abnormal glutamine metabolism is associated with clinical disorders such as osteoporosis and osteoarthritis and expected to provide novel guideline for treatments. In bone tissues, an integrated regulatory network where glutamine acting as the target participate BMSC differentiation, whereas researches of downstream effectors of glutamine metabolism are seldom studied currently. Therefore, the mechanism of glutamine in bone homeostasis is likely multifaceted and additional basic investigation is needed beyond doubt. Glutamine metabolism has diversified influences on other cells or tissues, for example, it impacted the cellular differentiation through the epigenetic regulation in embryonic stem cells [158]; nevertheless, it has not been elucidated in bone cells. Alternatively, glutamine supplement has been applied in some systemic disease treatment and is expected to restore the impairment of osteoporosis and osteoarthritis. Virtually, the targets of glutamine in bone disease therapy are little known. Therefore, more fundamental and clinical studies are needed to deeply investigate the role of glutamine metabolism in regulating bone homeostasis and provide a new strategy for the clinical treatment of bone diseases.

## Figures and Tables

**Table 1:** The application of glutamine in clinical trials.

	Disorders/treatment/diagnosis	Detailed effect
Digestive system disease	Postinfectious irritable bowel syndrome	Restore tight junction proteins, increase claudin-1 expression, and improve permeability [121]
	Crohn's disease	Increase the insoluble fraction of claudin-1 and occludin proteins, prevent the tight junction proteins, and maintain the intercellular junction [122]
	Short bowel syndrome	Provide energy for enterocytes, enhance the transport of sodium and water in the ileum, and upregulate intracellular protein synthesis [123–125]
	Acute pancreatitis	Improve lymphocyte proliferation, reduce proinflammatory cytokine, release C-reactive protein, and improve the nutritional status [126, 127]
	Cirrhotic	Increase blood ammonia [128]

Circulation system disease	Sickle cell disease	Raise the NAD redox ratio within sickle cells and synthesize NAD and decrease endothelial cell adhesion in sickled red cells [129, 130]
	Heart failure	Maintain a positive nitrogen balance and activate the suppressed oxidative metabolism [131]

Locomotor system disease	Duchenne muscular dystrophy	Inhibit whole-body protein degradation and stimulate insulin secretion [132, 133]

Systemic disorders	Critically ill patients	Maintain high level of HSP70 [134]
	Sepsis	Increase immune response, donate nitrogen for many anabolic processes, and promote wound healing [135]
	Type 2 diabetes mellitus	Delay gastric emptying to lower glycemia, stimulate GLP-1 concentration, and increase circulating insulin
	Low birthweight infants	Aid in maturation of the intestinal tract enhances growth, development, and function of the immunologic system [136, 137]

Imaging diagnosis	PET assay of tumor	A potential tumor biomarker for targeted radiotracer imaging [138]

Regulatory effect on certain treatments	Radiotherapy-induced toxicities	Protective effects of diarrhea minimized dermatitis [139, 140]
	Chemotherapy-induced toxicities	Treat neuropathy induced by vincristine and decrease mucositis severity [141–144]
	Peripheral blood stem cell transplantation	Improve CD3^+^ and CD4^+^lymphocyte recovery [145, 146]
	Liver transplantation	Synthesize glutathione and protect the liver graft against lipid peroxidation [147]
	Cardiac surgery	Enhance cell survival, attenuate the systemic inflammatory response, and prevent intracellular lactate accumulation [148, 149]
	Total knee replacement (TKA)	Suppress the loss of muscle strength after TKA [150]
